# Supplementary material for: Single‐domain antibodies targeting antithrombin reduce bleeding in hemophilic mice with or without inhibitors
Source: EMBO Mol Med. 2020 Mar 11;12(4):e11298. doi: 10.15252/emmm.201911298 (PMC7136963; doi:10.15252/emmm.201911298)
Supplement: Supplementary file 1 — Expanded View Figures PDF [file EMMM-12-e11298-s001.pdf]

Expanded View Figures

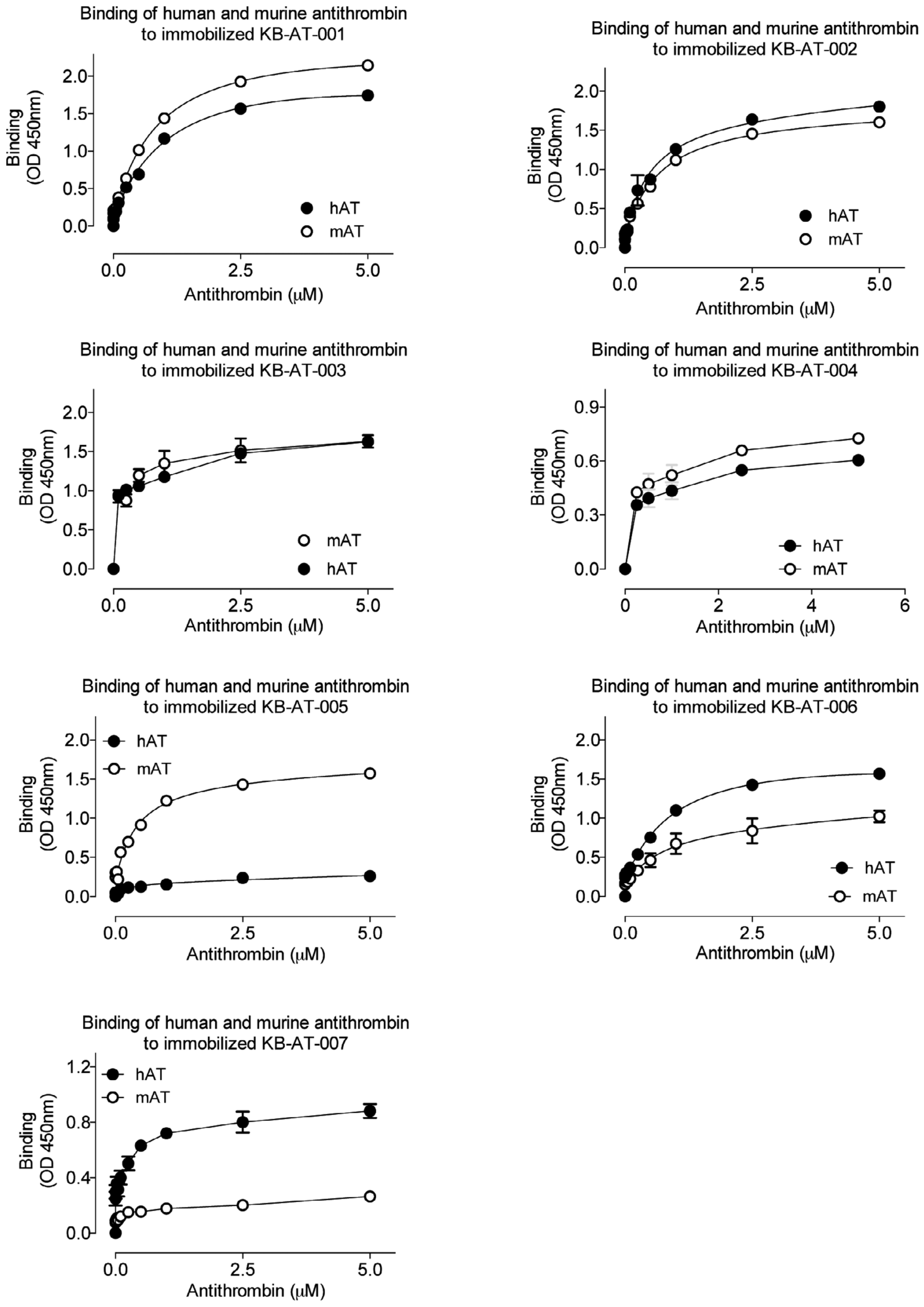

Figure EV1. Binding of human and murine antithrombin to immobilized monovalent sdAbs.

Human antithrombin and murine antithrombin (0–5 μM) were added to wells coated with monovalent sdAbs (5 μg/ml). Bound antithrombin was probed using polyclonal anti-antithrombin antibodies and detected via hydrolysis of 3,3′,5,5′-tetramethylbenzidine. Plotted is the observed OD at 450 nm versus the antithrombin concentration.

© 2020 The Authors
