# Supplementary material for: Single‐domain antibodies targeting antithrombin reduce bleeding in hemophilic mice with or without inhibitors
Source: EMBO Mol Med. 2020 Mar 11;12(4):e11298. doi: 10.15252/emmm.201911298 (PMC7136963; doi:10.15252/emmm.201911298)

Figure 4B

Lanes 1 and 3-7 are used in figure 4B in the manuscript.

**Sample name**
1-pSMD2 hAAT KB-AT 002/003
2-pSMD2 hAAT KB-AT 002/003 Sp0 (no HIS tag)
3-pSMD2 hAAT KB-AT 002/003 H1
4-pSMD2 hAAT KB-AT 002/003 H2
5-pSMD2 hAAT KB-AT 002/003 H4
6-pSMD2 hAAT KB-AT 002/003 H7
7-Untrasfected HepG2 cells
8-Cntrl +

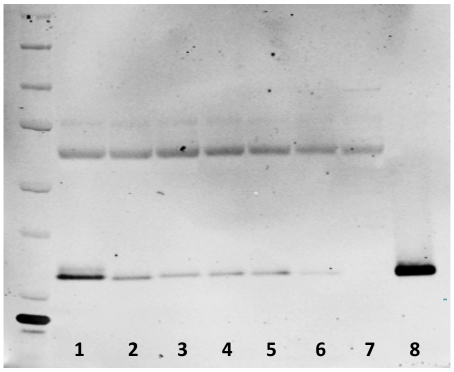

Supplement: Supplementary file 2 — Source Data for Figure 4 [file EMMM-12-e11298-s002.zip › EMM-2019-11298_Source_data_for_Figures_4B_uncropped_blots.pdf]
