# Supplementary material for: Single‐domain antibodies targeting antithrombin reduce bleeding in hemophilic mice with or without inhibitors
Source: EMBO Mol Med. 2020 Mar 11;12(4):e11298. doi: 10.15252/emmm.201911298 (PMC7136963; doi:10.15252/emmm.201911298)

Fig. 4C

Sample name
1 & 9 pSMD2 hAAT KB-AT 002/003
2 & 10 pSMD2 hAAT KB-AT 002/003 Sp0 (no HIS tag)
3 & 11 pSMD2 hAAT KB-AT 002/003 H1
4 & 12 pSMD2 hAAT KB-AT 002/003 H2
5 & 13 pSMD2 hAAT KB-AT 002/003 H4
6 & 14 pSMD2 hAAT KB-AT 002/003 H7
7 & 15 Untrasfected HepG2 cells
8 & 16 Cntrl + rec KB-AT-23

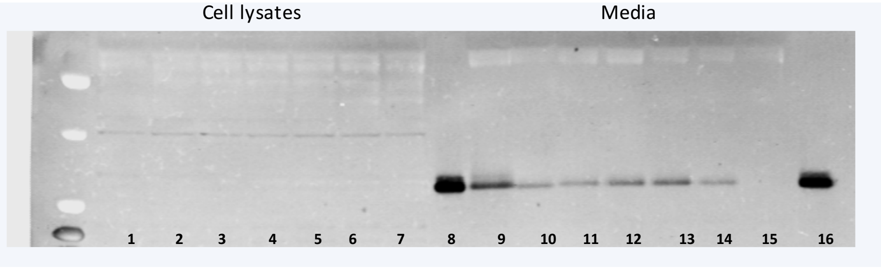

Supplement: Supplementary file 2 — Source Data for Figure 4 [file EMMM-12-e11298-s002.zip › EMM-2019-11298_Source_data_for_Figures_4C_uncropped_blots.pdf]
