# Supplementary material for: Single‐domain antibodies targeting antithrombin reduce bleeding in hemophilic mice with or without inhibitors
Source: EMBO Mol Med. 2020 Mar 11;12(4):e11298. doi: 10.15252/emmm.201911298 (PMC7136963; doi:10.15252/emmm.201911298)

Figure 5B

Lanes 11-4, lanes 9-11, lane 14 and lane 16 have been used.

Samples	FVIII KO n.	Groups
1	4	AAV8-KB AT 002/003 (low dose)
2	5	
3	6	
4	7	
5	11	AAV8-KB AT 002/003 (intermediate dose)
6	12	
7	13	
8	14	
9	24	AAV8-KB AT 002/003 (high dose)
10	25	
11	26	
12	27	PBS
13	31	AAV8-KB AT 002/003 (high dose)
14	32	
15	33	PBS
16	35	
17	Cntrl 100ng	
18	Cntrl 50ng	

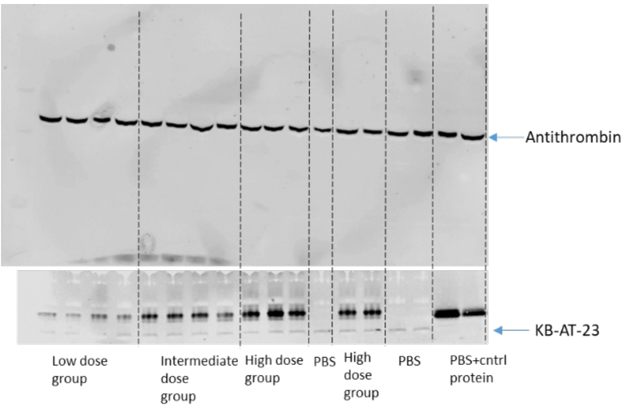

Supplement: Supplementary file 3 — Source Data for Figure 5 [file EMMM-12-e11298-s003.pdf]
