# Supplementary figures and images for: Single‐domain antibodies targeting antithrombin reduce bleeding in hemophilic mice with or without inhibitors
Source: EMBO Mol Med. 2020 Mar 11;12(4):e11298. doi: 10.15252/emmm.201911298 (PMC7136963; doi:10.15252/emmm.201911298)

Figure 6D

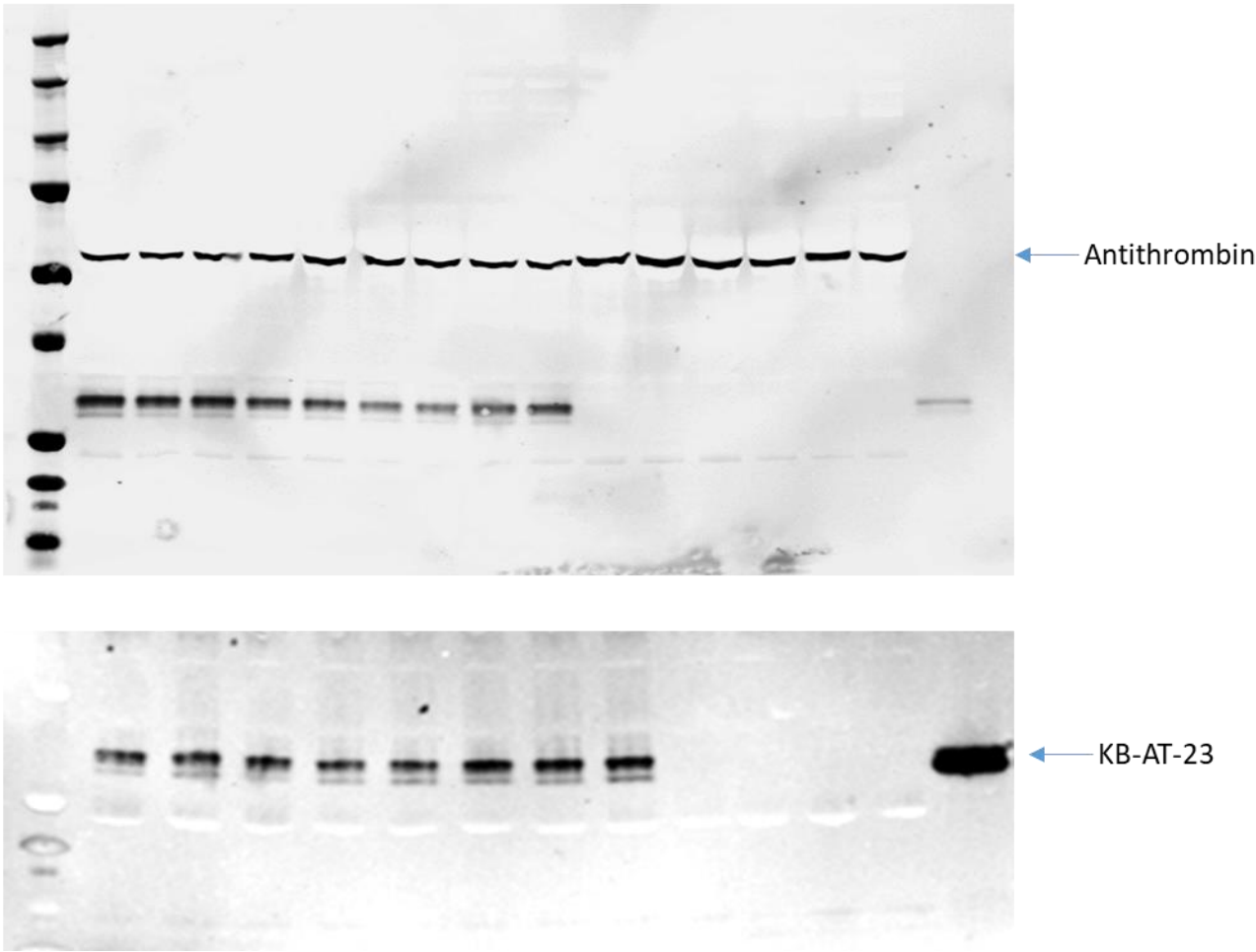

Supplement: Supplementary file 4 — Source Data for Figure 6 [file EMMM-12-e11298-s004.pdf]

**Figure 7C**

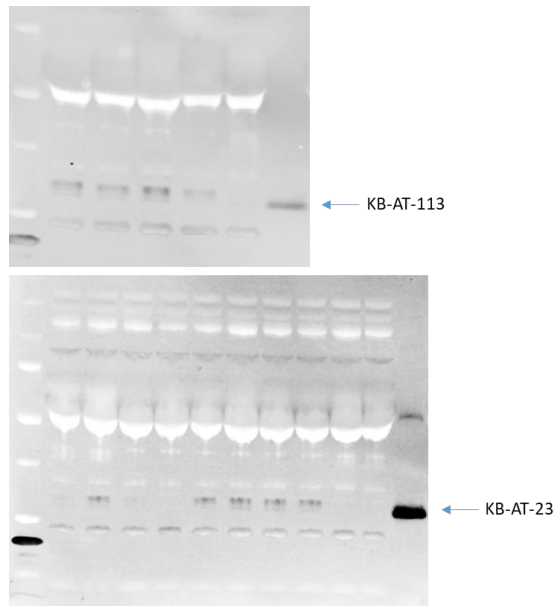

Supplement: Supplementary file 5 — Source Data for Figure 7 [file EMMM-12-e11298-s005.pdf]
